# Suitability of the Visual Field Index according to Glaucoma Severity

**DOI:** 10.5005/jp-journals-10008-1186

**Published:** 2016-02-02

**Authors:** Marina CC Sousa, Luis G Biteli, Syril Dorairaj, Jessica S Maslin, Mauro T Leite, Tiago S Prata

**Affiliations:** Resident, Department of Ophthalmology, Federal University of Sao Paulo, Sao Paulo, Brazil; Preceptor, Glaucoma Service, Department of Ophthalmology, Federal University of Sao Paulo, Sao Paulo, Brazil; Associate Professor, Department of Ophthalmology, Mayo Clinic, Florida, USA; Resident, Department of Ophthalmology and Visual Science, Yale University School of Medicine, CT, USA; Medical Staff, Glaucoma Service, Department of Ophthalmology, Federal University of Sao Paulo, Sao Paulo, Brazil; Assistant Professor, Department of Ophthalmology, Federal University of Sao Paulo, Sao Paulo, Brazil

**Keywords:** Functional correlation, Functional evaluation, Glaucoma blindness, Stages of glaucoma, Visual field index.

## Abstract

**Purpose:** To investigate the suitability of the visual field index (VFI) in different degrees of disease severity in glaucoma patients.

**Methods:** In this cross-sectional study, we consecutively enrolled patients with primary open-angle glaucoma and glaucoma suspects (ocular hypertension). All eyes required a reliable standard automated perimetry (SAP) test to be included. Subjects were categorized into five groups based on glaucoma severity using SAP’s mean deviation (MD). To evaluate the correlation among VFI, MD and pattern standard deviation (PSD), a linear regression model was built. To evaluate the nature of the correlation (i.e. linear *vs* nonlinear), results were plotted in a scatterplot graph.

**Results:** One hundred and twenty-two eyes of 81 patients (mean age, 59.8 ± 14.5 years) were included. A strong, positive association was found between MD and VFI values (R^2^ = 0.98, p < 0.001), showing a 3.2% reduction in the VFI for each dB loss in the MD index. It was noticed that 15% of eyes with mild glaucoma (average MD of -3.1 dB) had VFI > 99%. Considering only the eyes with mild and moderate damage in the regression, we found a weaker (nonlinear) correlation than the one we found using all eyes (R^2^ = 0.85, p < 0.001). There was also a significant, nonlinear correlation between VFI and PSD (R^2^ = 0.85, p < 0.001). Although higher PSD values were found with increasing visual field damage, this initial trend was reversed when VFI became smaller than 50%, approximately.

**Conclusion:** Visual field index had a strong correlation with MD; however, this correlation was weaker in mild disease, as some patients with early disease had very high VFI values (ceiling effect). Therefore, initial deterioration in visual field status (as assessed by MD values) in patients with early disease may not be detectable using the VFI alone.

**How to cite this article:** Sousa MCC, Biteli LG, Dorairaj S, Maslin JS, Leite M, Prata TS. Suitability of the Visual Field Index according to Glaucoma Severity. J Curr Glaucoma Pract 2015;9(3):65-68.

## INTRODUCTION

Glaucomatous optic neuropathy is characterized by loss of retinal ganglion cells with characteristic changes of the optic nerve head and visual field. Although several structural and functional tests have been developed to diagnose and monitor patients with glaucoma, standard automated perimetry (SAP) remains the most widely used method to access the visual deficit by glaucoma.^1^

Besides the analysis of graphs available in the printed examination of SAP, different indices have been developed to assist in the diagnosis and monitoring of patients with glaucoma. The most traditional are: the mean deviation (MD), usually related to generalized loss of function; the pattern standard deviation (PSD), more related to localized functional loss, and glaucoma hemifield test, which compare the functional loss in lower and upper hemifields.^2^ Bengtsson and Heijl introduced the visual field index (VFI), which is less affected by media opacity (as cataract) and takes into consideration the functional loss corrected for age spots identified as changed in the probability map of the PSD.^3^

These indices should be interpreted together in the evaluation of glaucomatous patients and may vary according to the type of field defect and the stage of disease. In this study, we investigated the suitability of the VFI and its correlations with the other available indices in different stages of primary open-angle glaucoma (POAG).

## METHODS

In this cross-sectional study, we consecutively enrolled POAG patients and glaucoma suspects (ocular hypertension) from the glaucoma sectors of the Federal University of Sao Paulo and Hospital Medicina dos Olhos, Brazil. The study was approved by the Institution’s Human Research Ethics Committee/Investigational Review Board and adhered to the guidelines of the Declaration of Helsinki. Written informed consent was obtained from all participants.

The medical records of all subjects were reviewed and a new SAP was performed (Humphrey 24-2). Visual field tests with unreliable results were not included in the analysis. Visual fields were considered reliable if they had no more than 33% of false negative responses, 15% of false positive responses, and less than 20% of fixation losses. Inclusion criteria were a diagnosis of POAG or ocular hypertension, with a history of three or more prior visual field tests. Patients with a history of previous ocular surgery, trauma or other ocular diseases (except cataract) were excluded.

Participants were divided into five groups according to the presence and severity of the disease, defined by their values of MD: group I (glaucoma suspects―ocular hypertension), group II (mild glaucoma, MD > -6 dB), group III (moderate glaucoma, MD between - 6 and - 12 dB), group IV (advanced glaucoma, MD between -12 and -20 dB), and group V (end stage glaucoma, MD < -20 dB).

The three global indexes (MD, PSD and VFI) were obtained and correlated using regression analyses. Their behaviors in the different stages of the disease were studied. Computerized analysis was performed using MedCalc Software (MedCalc, Inc., Mariakerke, Belgium), with a statistical significance set at p < 0.05.

**Table 1:** **Table 1:** Visual field indices of study patients according to disease stage

		*Suspects (n = 22)*		*Mild (MD < 6 dB;* *n = 54)*		*Moderate (-6 dB < MD* *< 12 dB; n = 16)*		*Advanced (12 dB < MD* *< -20 dB; n = 16)*		*End stage* *(MD > 20 dB; n = 14)*	
Age (years)		50.5 ± 10.0		57.5 ± 12.4		67.9 ± 9.4		65.7 ± 15.6		60.2 ± 17.0	
MD (dB)		–0.9 ± 1.1		–3.1 ± 1.7		–7.7 ± 1.7		–15.6 ± 2.2		–28.0 ± 3.9	
PSD (dB)		1.4 ± 0.4		3.5 ± 2.1		5.5 ± 2.3		11.6 ± 2.0		6.2 ± 3.9	
VFI (%)		99.3 ± 0.9		94.0 ± 4.4		84.1 ± 7.7		56.9 ± 6.8		13.4 ± 12.9	

MD: Mean deviation; PSD: Pattern standard deviation; VFI: Visual field index

## RESULTS

One hundred and twenty-two eyes of 81 patients (mean age 59.8 ± 14.5 years) were included. Of those tested, 44 were women (54%) and 37 were men (45%).

In group I (ocular hypertensive patients), the average of MD, PSD, and VFI were - 0.9 ± 1.1 dB, 1.4 ± 0.4 dB, and 99.3 ± 0.9% respectively. The mean age in this group was 50.5 ± 10.1 years. The mean age values of MD, PSD, and VFI in the different stages of the disease are described in Table 1.

**Graph 1:**
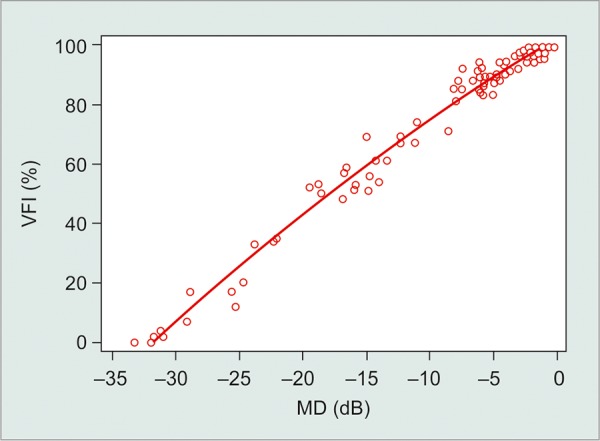
Correlation between VFI and MD (MD: Mean deviation; VFI: Visual field index)

**Graph 2:**
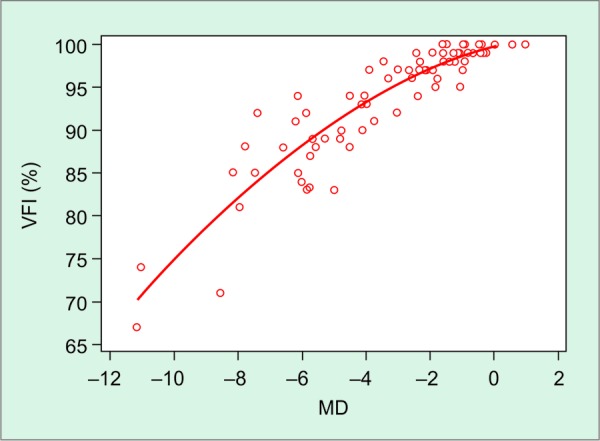
Nonlinear correlation between MD and VFI considering only the eyes with early and moderate functional damage (MD: Mean deviation; VFI: Visual field index)

A strong, positive association was found between MD and VFI values (R^2^ = 0.98, p < 0.001; Graph 1), showing a 3.2% reduction in the VFI for each dB loss in the MD index. In analyzing the correlation between MD and VFI, it was noticed that 15% of eyes with mild glaucoma (MD ranging between - 5.94 and - 0.26 dB, average of - 3.1 dB) had VFI greater than or equal to 99% (VFI average 94 ± 4.4%). In those eyes, the MD ranged from - 0.26 to - 2.18 (MD average of - 0.98 dB) and the PSD ranged from 1.34 to 2.6 dB (PSD mean of 1.6 dB). Considering only the eyes with mild and moderate damage in the regression, we found a weaker correlation than the one we found using all eyes (R^2^ = 0.85, p < 0.001; Graph 2). This may be a consequence of the very high VFI values observed in some patients with early damage.

**Graph 3:**
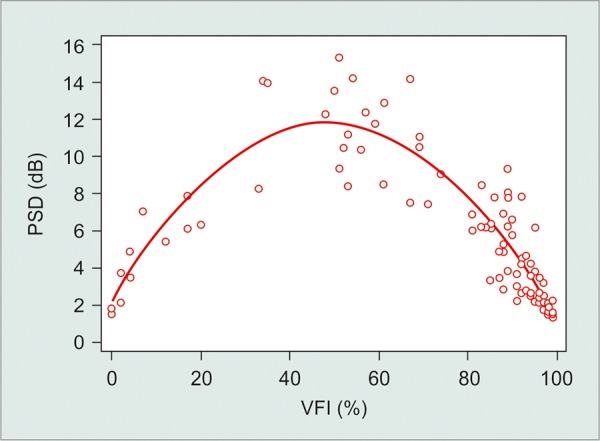
Nonlinear correlation between VFI and PSD (PSD: Pattern standard deviation; VFI: Visual field index)

**Graph 4:**
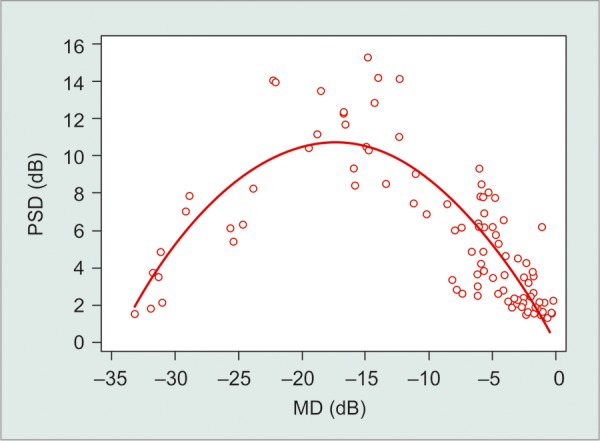
Nonlinear correlation between MD and PSD (PSD: Pattern standard deviation; MD: Mean deviation)

There were also significant, nonlinear correlations between VFI and PSD (R^2^ = 0.85) and MD and PSD values (R^2^ = 0.71; p < 0.001; Graphs 3 and 4). Higher PSD values were found with increasing visual field damage (as determined by MD or VFI). However, this initial trend was reversed with further functional damage (eyes with MD < - 17 dB or VFI < 50%, approximately).

## DISCUSSION

The functional evaluation of glaucoma patients by analyzing the visual field is essential to determine severity and progression of the disease. The VFI is expressed as a percentage of visual function, corrected for age. The index is calculated by considering the PSD for defects up to - 20 dB and the total deviation for more advanced visual field loss. The VFI was designed to be an intuitive index, describing the spectrum of visual field function from a normal of 100% to blindness at 0%. It has less influence of media opacity (such as cataract) and is more sensitive to central deficits in relation to MD.^2-7^ The present study shows that, in a clinical setting, although the VFI had a strong linear correlation with MD, this correlation was weaker in mild disease. Therefore, initial deterioration in visual field status (as assessed by MD values) in these patients may not be detectable using the VFI alone.

We noted a strong linear correlation between VFI and MD at different stages of glaucoma, except in mild glaucoma in which 15% of these eyes had VFI above or equal to 99% even with decreasing levels of MD. In most of these eyes (88%), the visual field loss had not reached the central visual field (no spot with significant reduction in sensitivity between the four paracentral points), which may have contributed to the VFI being so close to 100% in those eyes. Additionally, the logarithm nature of the visual field scale may compress values for initial glaucoma and expand these values for more advanced disease. For example, the loss of 1% of VFI when the patient has 100% represents a greater loss in light sensitivity than when the patient has a VFI of 50%. Our results corroborate those from Artes et al.^6^ The authors also reported a ceiling effect in eyes with initial functional damage (in 22% of those with MD > - 5.0 dB), with a linear correlation between VFI and MD when considering eyes presenting with worse visual field defects.^6^ A study by Dorairaj et al also investigated the correlation between VFI and MD in 75 eyes with moderate to advanced glaucoma (MD worse than - 12 dB).^8^ The correlation between MD and VFI was found to be excellent with R^2^ = 0.95.

Correlations between VFI *vs* PSD and MD *vs* PSD were not linear. This fact can be explained by the property of PSD to detect localized loss. The PSD is based on the pattern deviation plot, and as the defect becomes more diffuse, their values return to normal (toward zero). Thus, PSD is not a good parameter to monitor patients with advanced disease.^9^

We investigated correlations between the three indices in a single visual field. However, longitudinal studies have shown that the VFI is a good index to evaluate progression of the disease over time and is less affected by cataract than MD.^3,9^ Other studies suggest that VFI is possibly a greater tool for determining stability rather than for detecting glaucomatous progression.^10^ In our study, the usefulness of each index for monitoring disease progression was not determined.

Specific boundaries have been previously suggested to help to guide clinical decision-making. Heijl et al^11^ suggested an arbitrary cut-off of ‘half of the field ’ (MD -15 dB or worse), which would signify a significant decrease in quality of life. The correlation analysis of Dorairaj et al suggests that ‘half of field ’ corresponds to a VFI of 60% rather than 50%. This finding suggests that clinicians should try to keep their glaucoma patients in the upper 40% of the VFI throughout the course of the disease. Another landmark, which is utilized by the United States Department of Social Service, is MD - 22 dB is equivalent to a central field of less than 20°. This defines a legally blind field. Based on our findings, this would correspond to a VFI value of approximately 33%. Using these mathematical correlations, we expect to provide clinicians with VFI values (in percentage) to help to denote glaucoma severity landmarks, which we believe are more intuitive than the typically used MD values.

Our study has some limitations. First, the relatively young age of our patients (59.8 ± 14.5 years) and, consequently, the lower prevalence of cataracts, may have contributed to the strong correlations we found between MD and VFI values. Second, as it was a cross-sectional study, we did not investigate the correlations between the different indices in disease progression. Finally, the variability of each index and possibly associated factors were not accessed.

In conclusion, despite being a relatively new index based mainly in the pattern deviation chart, the VFI correlated linearly with the well-established MD in patients with moderate and advanced disease. However, this correlation was weaker in mild disease, as some patients with early disease had very high VFI values (ceiling effect). In this group, the diagnostic sensitivity may be diminished. The interpretation of these indices should be made jointly considering its limitations and quirks.
